# Plant Trait Diversity Buffers Variability in Denitrification Potential over Changes in Season and Soil Conditions

**DOI:** 10.1371/journal.pone.0011618

**Published:** 2010-07-16

**Authors:** Bonnie M. McGill, Ariana E. Sutton-Grier, Justin P. Wright

**Affiliations:** Duke University, Durham, North Carolina, United States of America; University College London, United Kingdom

## Abstract

**Background:**

Denitrification is an important ecosystem service that removes nitrogen (N) from N-polluted watersheds, buffering soil, stream, and river water quality from excess N by returning N to the atmosphere before it reaches lakes or oceans and leads to eutrophication. The denitrification enzyme activity (DEA) assay is widely used for measuring denitrification potential. Because DEA is a function of enzyme levels in soils, most ecologists studying denitrification have assumed that DEA is less sensitive to ambient levels of nitrate (NO_3_^−^) and soil carbon and thus, less variable over time than field measurements. In addition, plant diversity has been shown to have strong effects on microbial communities and belowground processes and could potentially alter the functional capacity of denitrifiers. Here, we examined three questions: (1) Does DEA vary through the growing season? (2) If so, can we predict DEA variability with environmental variables? (3) Does plant functional diversity affect DEA variability?

**Methodology/Principal Findings:**

The study site is a restored wetland in North Carolina, US with native wetland herbs planted in monocultures or mixes of four or eight species. We found that denitrification potentials for soils collected in July 2006 were significantly greater than for soils collected in May and late August 2006 (*p*<0.0001). Similarly, microbial biomass standardized DEA rates were significantly greater in July than May and August (*p*<0.0001). Of the soil variables measured—soil moisture, organic matter, total inorganic nitrogen, and microbial biomass—none consistently explained the pattern observed in DEA through time. There was no significant relationship between DEA and plant species richness or functional diversity. However, the seasonal variance in microbial biomass standardized DEA rates was significantly inversely related to plant species functional diversity (p<0.01).

**Conclusions/Significance:**

These findings suggest that higher plant functional diversity may support a more constant level of DEA through time, buffering the ecosystem from changes in season and soil conditions.

## Introduction

Denitrification in wetland soils is an important ecosystem service that removes nitrogen (N) from N-polluted watersheds, buffering soil, stream and river water quality from excess N by returning N to the atmosphere before it reaches lakes or oceans [1]. Excess N delivered to estuaries and oceans produces harmful algal blooms, which can lead to hypoxia and even dead zones resulting in widespread fish kills [2], [3]. Eutrophication, caused by excess inputs of nutrients including N, is a leading problem facing US coastal waterways which is likely to worsen as human use of inorganic fertilizers and fossil fuels continues to increase [4]. Here we investigate the controls on the variability in the activity of denitrifying bacteria responsible for this critical ecosystem service.

Denitrification is an anaerobic process that reduces nitrate (NO_3_^−^), producing mainly the gases dinitrogen (N_2_) and nitrous oxide (N_2_O)—a greenhouse gas [5]. In order to reduce NO_3_^−^ denitrifying bacteria (denitrifiers) also require organic carbon (OC) as an energy source and are typically most active near the “hot-spot” interface with the oxic zone where nitrification occurs [6], [7]. With such strict requirements, denitrification rates are highly spatially and temporally variable [6], [8]. Therefore a measure of the denitrifiers' *potential* ability to denitrify under optimal conditions yields more information about denitrifier functioning than *in situ* denitrification measurements—where the denitrification rate is a product of the availability of denitrification substrates in the soil environment and not a measure of microbial community's functional potential. The Denitrification Enzyme Activity (DEA) assay is a widely used method for measuring denitrification potential [9] that provides soil slurries with excess OC and NO_3_^−^ under anoxic conditions and measures N_2_O production over a short incubation period [9]. The rapid 90 minute incubation simulates the microbes' response to a “hot moment” [6] and can be viewed as measuring the functional capacity of the microbial community as opposed to a measurement of *in situ* denitrification where the denitrification rate may be limited by substrate availability, redox status, and/or temperature.

Since the DEA assay removes constraints on microbial functioning, denitrification potential is often thought to be more constant over time than *in situ* denitrification rates [5]. This assumes that either microbial communities are constant over time or that communities can respond to ideal conditions in the relatively short time scales of incubations and hot moments. However, some studies have shown that denitrification potential can vary over time at a site [10], [11], [12], [13]. The key factors controlling this variability in the function of the microbial community have not been fully explored. Soil NO_3_^−^ and OC availability as well as soil redox status are known to be important factors in *in situ* denitrification rates [14]. In DEA rate measurements these factors are not limiting because they are specifically supplied in excess, while the microbial community response to these ideal denitrification conditions is shaped by the field conditions in which the microbial community developed. Therefore, field levels of NO_3_^−^ and OC are likely to be related to, but not limiting, the microbial community response measured by DEA. Thus, plant diversity which could affect soil microbial activity, NO_3_^−^ and OC, could influence DEA rates as well.

Plant diversity has been shown to have strong effects on microbial communities and belowground processes [15], [16] and could potentially alter the functional capacity of denitrifiers. Zak et al. [17] reported that greater plant diversity led to higher levels of N mineralization, which could lead to higher denitrification potential. A large number of studies have shown that greater plant diversity leads to greater primary productivity (for a review of these see [18]), which could translate to greater belowground C and N inputs, stimulating the denitrifier community. While these and other studies have described the relationship between plant functional diversity (FD), root processes, soil properties, and soil microbes, this is one of the first studies with experimental evidence linking plant functional diversity and soil denitrification. If different species of plants have different seasonal impacts on soil OC and N inputs—and, thus, microbial communities—then greater plant FD might be predicted to stabilize the microbial community leading to less variance in denitrification potential over time. Similarly, the “insurance effect” [19] and “portfolio effect” [20] predict that biodiversity buffers ecosystem functions from environmental changes when different species respond differently to environmental variability. In terms of denitrification, this could mean a more diverse community of plants maintains higher productivity despite environmental changes, which could provide more consistent inputs of plant C belowground. Both the stimulatory belowground and insurance/portfolio effects could produce more consistent levels of denitrification substrates and may lead to less variance in denitrification potential over time.

Here we explore temporal patterns of variability of denitrification potential in a restored wetland. We tested the denitrifier community's functional response to differences in environmental conditions and plant community diversity. The three questions of this study are: (1) Does DEA vary through the growing season? (2) If so, can we predict DEA variability with environmental variables? (3) Does plant functional diversity affect DEA variability?

## Methods

### Study site and experimental design

The study site is located in a restored riparian wetland in the Duke University Stream and Wetland Assessment Management Park (SWAMP), located along Sandy Creek in the Duke Forest in Durham, NC (36° 00' N, 78° 54' W). Soils in this area are primarily Cartecay silt loams and Mayodan sandy loams [21]. Restoration took place in 2005, and in the process all riparian vegetation was removed and the site was graded to a constant elevation. Fifty-one 2×2 meter plots were planted in May 2005 with a total of 100 seedlings added as plugs in mixes of one, four, or eight species from a pool of ten species: *Carex crinita*, *Carex lurida*, *Scirpus cyperinus*, *Juncus effusus*, *Panicum virgatum*, *Chasmanthium latifolium*, *Eupatorium fistulosum*, *Vernonia noveboracensis*, *Asclepias incarnata*, and *Lobelia cardinalis*. In the plots with four species (15 plots), 25 individuals of each randomly selected species were planted. In the plots with eight species (16 plots), either 12 or 13 individuals of each randomly selected species were planted. The species in the study were selected from a list of recommended species for North Carolina stream restoration [22] based on commercial availability and to maximize trait diversity. One, two, or three monoculture plots of each species were planted; however due to high herbivory on two of the species' monocultures, only eight species' monocultures were used in these analyses for a total of 15 monocultures.

### Soil sampling and laboratory analysis

Soil samples were collected in the second growing season of the experiment, 2006, in early May, mid-July, and late August. Two soil samples (2.5 cm diameter) from each plot were collected in plastic sleeves from the upper 15 cm of each plot using a piston corer. These samples were stored on ice until arrival at the lab then stored at 4°C until they could be analyzed, typically within a week of collection. Upon arrival at the laboratory, the two cores from each plot were bulked together and sieved through a 4.75 mm sieve prior to analysis. To determine soil redox status, we measured percent soil moisture by oven-drying a sub-sample of each soil at 105°C for 24 hours. A sub-sample of this dried soil was then used to determine percent soil organic matter (OM) by loss on ignition at 450°C [23]. Total Inorganic N (TIN) was measured with two replicate 3 g field-moist sub-samples from each plot that were analyzed for 2 M potassium chloride (KCl) extractable nitrate + nitrite (NO_3_^−^ + NO_2_^−^) and ammonium (NH_4_^+^) [24] on a Lachat QuikChem 8000 (Lachat Instruments, Loveland, CO, USA). We chose to sum NO_3_, NO_2_^−^, and NH_4_^+^as TIN because during most of the growing season these wetland soils remained relatively oxic, which allows soil N to readily cycle between NO_3_^−^ and NH_4_^+^. Therefore, TIN serves as a more integrated measure of soil N available to the microbial community than NO_3_^−^ alone. We determined microbial biomass C (MBC) using a modified version of the Voroney and Winter [25] chloroform incubation technique. For each plot, four replicate 5 g field-moist sub-samples were prepared, two of the replicates were unfumigated and two were fumigated. We applied 0.5 mL of chloroform to cotton balls in the headspace of the fumigated sample containers and the samples incubated for seven days in the dark before they were extracted with 0.5 M potassium sulfate (K_2_SO_4_). Non-fumigated samples were extracted immediately. We analyzed unfumigated and fumigated samples for Total Organic Carbon (TOC) on a Shimadzu 5000 TOC analyzer (Kyoto, Japan) and calculated microbial biomass as the difference between the fumigated and unfumigated values of TOC [26]. The replicates were then averaged for each plot.

The DEA assay [5], [27] was used as an index of denitrification potential. DEA is a measure of potential denitrification because C and N are supplied in excess and the incubation is carried out under anaerobic conditions. Simultaneously, DEA utilizes the acetylene (C_2_H_2_) block technique to inhibit formation of the end product N_2_, such that N_2_O gas produced is a function of the level of enzyme in the sample [5]. In the lab, duplicate 5 g samples of homogenized, field-moist soil were amended with 10 mL of a solution of 0.5 g/L dextrose and 0.72 g/L potassium nitrate (KNO_3_) to ensure non-limiting substrate conditions, and 0.125 g/L chloramphenicol to inhibit protein synthesis. The slurries were made anaerobic by repeated flushing with N_2_ gas. Flasks were vented with a needle followed by an injection of 10 mL of acetylene into each flask [27], [28]. Gas samples were collected at 0, 30, 60, and 90 minutes with a syringe. Flasks were placed on an orbital shaker at 125 rpm for the 90 minute incubation. Gas samples were stored in evacuated glass vials until analysis (max holding time 48 hours) on a Shimadzu GC-17A ^63^Ni electron capture detector gas chromatograph (Shimadzu, Inc., Columbia, MD, USA.). N_2_O dissolved in the slurry was corrected with the Bunsen equation [29]. We used the maximum activity measured during the incubation to calculate the linear DEA rates.

In an effort to standardize our DEA rates, we calculated *mass-standardized DEA*, which is the DEA rate per unit MBC. Groffman and Tiedje [30] used mass-standardized DEA to study variability in denitrification. Since MBC is a useful measure of C availability, variation in it can point to differences in denitrification among ecosystems [30]. Groffman and Tiedje found that mass-standardized DEA was a stronger predictor of annual denitrification N loss than DEA alone [30]. We acknowledge that MBC is a measure of all microbes, only some of which are denitrifiers; however the advantage of mass-standardized DEA is that it allows for a more accurate comparison of samples by measuring the denitrification potential per unit microbial biomass rather than comparing simply denitrification rates where variability may be confounded by differences in the size of the microbial population as well. A change in mass-standardized DEA over time provides stronger evidence, versus DEA rates alone, that the denitrifier community's functional potential is shifting. Here we will examine both DEA and mass-standardized DEA rates, because the former is the most widely reported and the latter provides a better metric for comparison among treatments.

### Functional Diversity (FD) calculation

We used a multivariate, trait-based metric of plant functional diversity (Petchy's FD) that includes traits that are assumed to be functionally important, meaning these traits inform about the differences between species that affect the target ecosystem function [31]. Plants affect denitrification indirectly through inputs to soils that influence the microbial environment [32], [33]. In order to select traits appropriate for a denitrification FD calculation, we considered three pathways by which plants are known to influence denitrification via soil inputs: C quality, C quantity, and the redox status of the soil. Prior to statistical analyses, we selected traits related to each category.

Both the quality and quantity of soil C have been shown to limit denitrification [14], [34], [35], [36], [37]. For plant C quality inputs we measured: (1) C:N ratio of roots and (2) C:N ratio of aboveground biomass from individuals of each species grown in the greenhouse. Ideally all trait measurements would have been on individuals growing in the field; however, we were unable to measure all traits in the field due to the destructive nature of these trait measurements. (See the Discussion for more on trait measurements on field versus greenhouse individuals.) Greenhouse individuals of each species were started from seed in March 2005, transplanted to individual pots as seedlings in early April 2005, and grown in a standard greenhouse potting mix (Metromix). Species were then grown in pots for two months under greenhouse conditions designed to replicate temperature, humidity, and photoperiod at the field site. Species were kept at a constant water level with saturation at 15 cm below the soil surface. C:N ratios of leaves and roots were measured on a FlashEA 1112 Elemental Analyzer (Thermo Scientific, Waltham, MA, USA). For plant C quantity inputs we measured (1) aboveground biomass, which was calculated as the average aboveground biomass harvested from the two field monocultures of each species in September 2006 and (2) root biomass from the monocultures' soil cores. The quantity of plant soil C inputs can directly and indirectly stimulate denitrification: directly by providing necessary C substrates for microbial metabolism of NO_3_^−^ and indirectly by priming the soil for decomposition of organic material, increasing N mineralization and available NO_3_^−^ [38], [39].

A third pathway by which plants may affect denitrification is through modification of the redox conditions in the soil via delivery of oxygen through radial oxygen loss. In an anaerobic wetland environment, root porosity facilitates nitrification by allowing oxygen to be released from the roots [40]. Available soil nitrate and rates of nitrification have both been found to be tightly related to denitrification [14], [41], [42]. We measured root porosity using the pycnometer method [43] on roots from greenhouse individuals to estimate the amount of oxygen that could be transported through the root system of each plant to the soil.

These five traits—C:N ratios of roots and aboveground biomass, root and aboveground biomass, and root porosity—were used to calculate individual measures of plant trait diversity, FD, for each plot. These traits were transformed into standard deviation units, z-scores, so that all traits would be equally weighted no matter their original units. These z-scores were used to calculate dendrograms of the ten planted species, with a calculated branch length for each species based on the five traits indicating how different each species was from the others. A longer branch length means a species is more different from the rest of the species. To calculate the final FD for each plot, the branch lengths for each species present in a plot were summed. FD scores ranged from 3.72 to 14.08. See Petchey and Gaston [44] for a more detailed description of the FD calculation. Species were considered to be present in a plot when their biomass accounted for at least 10% of the total plot biomass. This meant that for the FD calculations plots had between 1 and 8 species present.

### Statistical analyses

To determine whether DEA, mass-standardized DEA, and soil variables exhibited significant variation across sampling dates, we used ANOVAs and Tukey's HSD post-hoc test. We tested the relationship of both DEA and mass-standardized DEA with soil variables and FD using a General Linear Model (GLM) with normal error distribution for each sample date. We calculated the coefficient of variation (CV) of the three sample dates' values for DEA, mass-standardized DEA, and the soil variables to quantify variability through time. Another GLM was used to compare the CV of DEA with the CV of the soil variables and FD. This GLM was repeated replacing DEA with mass-standardized DEA. MBC was removed from the latter models since it is part of the calculation of mass-standardized DEA. TIN data were log transformed. Plot level FD values do not have a CV since it was not measured at each sample date. Statistical analyses were performed using JMP 7.0 (SAS Institute, 2007).

## Results

**Figure 1:**
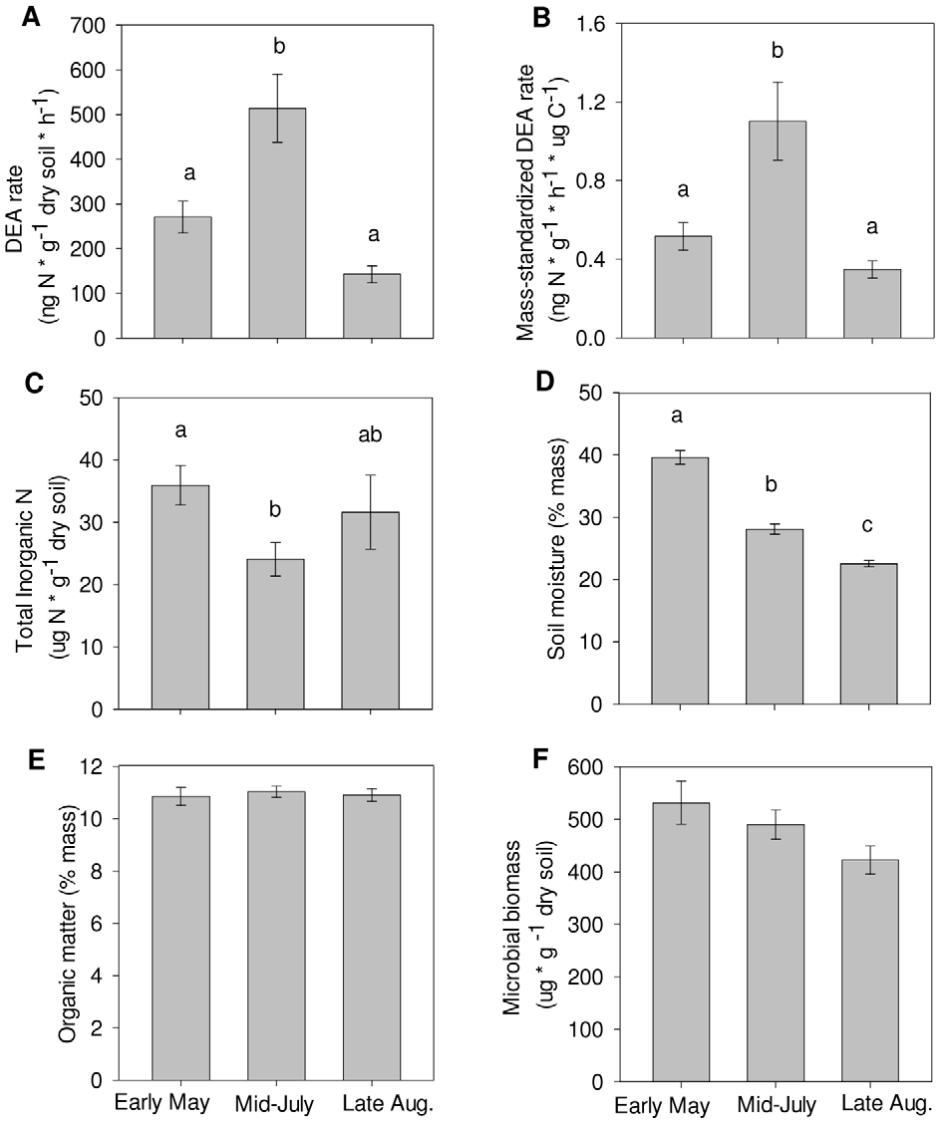
DEA rates and soil variables over time. Panels: A) DEA (df = 2, 134; *F* = 14.15; *p<*0.0001), B) Mass-standardized DEA (df = 2, 134; *F* = 10.11; *p<*0.0001), C) Total Inorganic N (df = 2, 133; *F* = 5.88; *p* = 0.0036), D) Soil moisture (df = 2, 134; *F = *107.52; *p*<0.0001), E) Organic matter (df = 2, 134; *F* = 0.12; *p = *NS), F) Microbial biomass C (df = 2, 134; *F* = 2.78; *p = *NS). Error bars indicate standard error. Bars with letters that do not match indicate a significant difference measured using an ANOVA and Tukey's HSD post-hoc test.

Both DEA (df = 2, 134; *F* = 14.15; *p<*0.0001; Figure 1A) and mass-standardized DEA (df = 2, 134; *F* = 10.11; *p<*0.0001; Figure 1B) varied significantly by sample date, with significantly higher values in mid-July than early May and late August. Among the explanatory variables, both TIN (df = 2, 133; *F* = 5.88; *p* = 0.0036; Figure 1C) and soil moisture (df = 2, 134; *F* = 107.52; *p*<0.0001; Figure 1D) showed significant variation over time with the highest levels of both TIN and soil moisture in May. Organic matter and MBC (Figures 1E and 1F) did not vary over time.

The explanatory variables that predicted DEA rates in the GLM of each sample date shifted over the course of the growing season (Table 1 and Figure S1). In May TIN was significantly related to DEA; in July OM was significantly related to DEA; and in August both OM and FD were significantly related to DEA. When we repeated these analyses using mass-standardized DEA (Table 1 and Figure S2) instead of DEA, variables predicting mass-standardized DEA differed from those predicting DEA in all months except May in which TIN was the only significant predictor of both mass-standardized DEA and DEA. (In May soil moisture was nearly significant in the mass-standardized DEA model). In July none of the explanatory variables were significantly related to mass-standardized DEA, and in August both TIN and FD were significant predictors of mass-standardized DEA.

**Table 1:** General Linear Model results testing for the ability of explanatory variables to predict DEA rates and mass-standardized DEA rates at each sample date.

Dependent variable	Explanatory variable	Early May Coeff.	Early May *p* 1	Mid-July Coeff.	Mid-July *p* 1	Late Aug. Coeff.	Late Aug. *p* 1
*DEA rates* 2	Plant functional diversity	11.74	0.22	−3.28	0.85	10.44	**0.03**
	Total Inorganic N	479.90	**<0.01**	372.84	0.12	25.94	0.63
	Microbial biomass C	0.03	0.86	−0.20	0.60	0.06	0.52
	Organic matter	26.14	0.16	154.71	**0.02**	46.48	**<0.001**
	Soil moisture	−3.59	0.58	14.69	0.40	0.48	0.92
*Mass- standardized DEA rates* 3,4	Plant functional diversity	0.02	0.35	−0.02	0.53	0.03	**<0.01**
	Total Inorganic N	1.17	**<0.01**	0.78	0.09	0.38	**<0.01**
	Organic matter	0.02	0.68	0.04	0.72	0.01	0.64
	Soil moisture	−0.03	**0.05**	0.06	0.10	−0.01	0.33

1 Bold *p-*values indicate significance (*p*<0.05).

2 For the early May and mid-July DEA models *n* = 46 and late August *n = *44.

3 For the early May mass-standardized DEA model *n* = 46, mid-July *n* = 45, and late August *n = *44.

4 MBC is not included as an independent variable because it is part of the mass-standardized DEA calculation.

**Figure 2:**
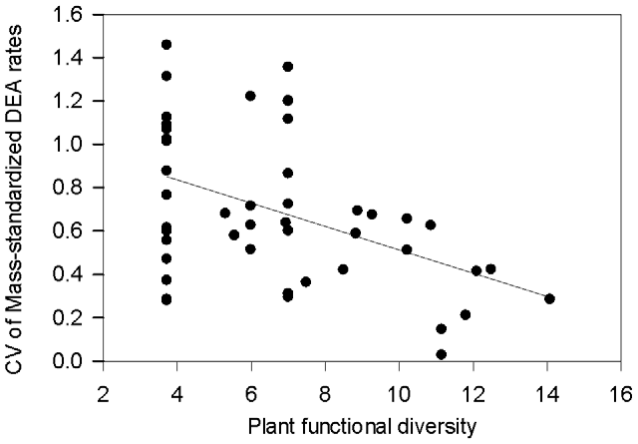
The relationship between the CV of mass-standardized DEA rates and plant functional diversity. Scatter plot of the CV of mass-standardized DEA rates in early May, mid-July, and late August 2006 from each plot versus the FD value for that plot (*n* = 46, *p* = 0.0012, R^2^ = 0.20). Regression analyzed using a general linear model. Each point represents one plot.

While the relationship between the CV of DEA and FD was not significant (*n* = 46, *p* = 0.28, Table 2, and Figure S3), the CV of mass-standardized DEA decreased significantly as plot FD increased (*n* = 46, *p* = 0.0012, Table 2, and Figure 2). To examine whether particular species' effects on DEA might be driving this FD effect, we looked at the DEA patterns in monoculture plots. There was no significant species or date-by-species interaction among the monoculture plots, i.e. different species did not appear to promote different patterns in DEA over time (Figure S4).

**Table 2:** General Linear Model results testing for the ability of the coefficient of variation (CV) of the explanatory variables over the three sample times to predict the CV of the DEA and mass-standardized (m-s) DEA rates over the same sample times.

Explanatory variable	CoefficientCV of DEA	*p* 1 CV of DEA	Coefficient CV of m-s DEA	*p* 1 CV of m-s DEA
Functional diversity^2^	−0.02	0.24	−0.06	**<0.01**
CV Total Inorganic N	−0.08	0.69	0.01	0.97
CV Microbial biomass C^3^	−0.91	0.14	–	–
CV Organic matter	−0.01	0.99	0.40	0.53
CV Soil moisture	0.09	0.87	0.39	0.48

Model coefficients and *p*-values are reported.

1 Bold *p-*values indicate significance (*p*<0.05).

2 A CV cannot be calculated for functional diversity since it was not measured at each sample date.

3 The CV of MBC is not included as an independent variable in the CV of mass-standardized DEA rates model because it is part of the mass-standardized DEA calculation.

## Discussion

The main objective of this study was to determine whether DEA varied through time and if so, whether environmental factors or plant diversity could explain the variability. We determined that (1) DEA varied through time; (2) the environmental factors related to DEA shifted through time; and (3) plant FD had a significant effect on mass-standardized denitrification variability through time. These results suggest that the functional capacity of the denitrifier community is shifting over time. Environmental factors alone did not predict denitrification potential consistently through time, while higher levels of plant trait diversity were associated with reduced variability through time.

### DEA variability through time

Typically the microbial process of denitrification is viewed as simply a function of environmental factors (e.g. C, N, and redox status) in the biogeochemical model of denitrification [45]. Denitrification is one of the most widely distributed microbial processes, second only to aerobic respiration [46]. As a result, it is easy to assume that denitrifier community dynamics do not impact denitrification, yet this assumption has not been carefully tested [45]. We measured denitrifier community functioning using the DEA method, which controls for the effect of environmental variables by supplying the microbial community in the sample with an excess of the required denitrification substrates, NO_3_^−^ and OC, and by making the environment anoxic. A change over time in DEA rates can be an indicator of a change in microbial community function in response to changes in the soil environment and/or plant trait diversity.

Both DEA and mass-standardized DEA rates were significantly higher in mid-July than in early May or late August (Figures 1A and 1B). Several papers describe a seasonal pattern of *in situ* denitrification [46], [47], [48], [49], [50], but we are aware of only a handful of studies that report DEA rates over time [10], [12], [13]—all observed different patterns of seasonal variation. Parsons et al. [10] and Strauss et al. [12] measured *in situ* denitrification and DEA and observed strikingly different seasonal patterns between the two metrics. Parsons et al. [10] found a significant positive correlation between DEA and soil moisture at their lowland site over one year. The Strauss et al. [12] study is one of two studies that described a seasonal pattern in DEA similar to the peak in mid-summer that we observed. They measured DEA and an unamended DEA—no nutrients added—over a two year period. The unamended DEA rates peaked in winter while the amended DEA rates had a significant peak in summer. The second study was by Wallenstein et al. [13]; they observed a peak in DEA rates in mid-summer in N-fertilized plots. These studies, in addition to this study, show that DEA changes with time and site, so it is clear that denitrifier community dynamics do have an impact on denitrification potential. Despite the aforementioned studies' reports of seasonal patterns in DEA, none have aimed to link seasonal variation in DEA to specific factors, which we will discuss in the next two sections.

### Denitrification and soil variables

Although it is understood that *in situ* denitrification varies over time due to changes in environmental factors including soil redox status and N and C availability, it has been assumed that DEA variation can be explained by these same factors [5]. Therefore, it is surprising that the soil variables measured in this study were tightly related to DEA and mass-standardized DEA rates only at single time points and not consistently over the three sampling dates (Table 1 and Figures S1 and S2). We found that DEA increased from May to July (Figure 1A) as field levels of inorganic N dropped significantly (Figure 1C), perhaps as a result of denitrification, plant uptake, or decreased inputs of N to the wetland from the watershed. The drop in DEA from July to August was not paralleled by a drop in field levels of TIN. The DEA assay is not limited by soil N since the assay provides N in excess. However, soil N levels could limit or influence the microbial community composition or biomass, which could affect the level of denitrification enzymes in the soil and therefore the denitrification potential. Other studies have shown that DEA rates are affected by soil moisture and soil N levels [51], but we did not find that this relationship between the changes in soil variables and DEA *over time* was significant or consistent. These results suggest that changes in DEA over time cannot be explained by any one factor; rather the environmental controls on the denitrifier community are complex and can shift within a single growing season.

### Denitrification and plant functional diversity

Plant FD was a significant predictor of mass-standardized DEA variability where plots with higher FD tended to have lower mass-standardized DEA variability (Table 2 and Figure 2). This finding corresponds with the “insurance effect” [19] and “portfolio effect” [20], both of which suggest that if species' performances are not positively correlated over time, greater diversity should lead to decreased temporal variance. Numerous studies have shown that higher plant diversity leads to increased productivity (for a review of these see [18]), and it is possible that plots with higher plant functional diversity had higher levels of below-ground productivity and C exudation. These inputs could promote denitrification throughout changing environmental conditions, so that the denitrification in polycultures is less variable than in monocultures, thus dampening temporal variability in mass-standardized DEA in plots with high plant FD. Similarly, Sutton-Grier et al. [52] found that greater plant FD led to greater denitrification potential at plots at this same study site, but mainly in plots with higher ambient levels of soil resources.

Given the observed FD effect on denitrification potential, we expected to see differences in DEA rates among the monocultures; but surprisingly peak DEA rates were similar for all species in monocultures and the highest overall rates of DEA were found in July in most of the monocultures (Figure S4). This indicates that individual plant species grown in monoculture were not stimulating denitrification potential at different times during the growing season. One resolution to this puzzling result is that in polycultures these species may take on complementary phenological niches when competing for the same resources. Thus, the FD effect on denitrification potential in a polyculture may be greater than the sum of its parts.

While this is one of the first studies with experimental evidence linking plant functional diversity and soil denitrification, other studies have described the relationship between plant FD, root processes, soil properties, and soil microbes. Zak et al. [17] showed that greater plant diversity altered soil microbial community composition and activity via increased plant productivity. Dybzinski et al. [15] further tested the diversity effect on plant productivity by investigating the fertility of soil beneath plots of varied levels of diversity and found that more diverse plots can support increased productivity via greater nutrient retention and inputs. Fornara et al. [16] found increased root N release and soil N mineralization in plots with complementary plant functional diversity. Collectively, these studies indicate that plant communities and plant diversity have important influences on soil microbial processes.

Given the importance of plant traits and communities on microbial processes, one promising avenue for future research should address how plant trait phenotypic plasticity impacts ecosystem function. Studies have shown that both abiotic and biotic factors can influence plant phenotypic plasticity, but the consequences of phenotypic plasticity on plant community dynamics are not well understood [53]. Therefore, a better understanding of phenotypic plasticity, including in which species and under what conditions it occurs, could be a productive and useful avenue for future research to help solidify our understanding of the role of plant traits in ecosystem dynamics.

### Conclusions

The seasonal DEA variation documented here indicates a change in the denitrifier community's ability to respond to resource pulses—meaning the ability of the microbial community to denitrify—is shifting over time. Our results suggest that rather than using DEA to get a single snapshot of soil microbial community functioning, we can use it as a tool to track changes in microbial community functioning in response to changes in environmental conditions through time. Researchers should consider these implications for planning and interpreting DEA assays. Although we found soil variables were related to DEA rates at single sampling times, none of them consistently predicted DEA through time despite being known as important factors in the denitrification process. The fact that we found that variation over time in DEA rates was not explained by changes in soil variables bolsters the need for microbial community dynamics to be included in biogeochemical models [45], and there is a growing body of evidence for this [54]. Our exploration of factors controlling denitrifier community functioning could provide additional evidence to include such information in biogeochemical models. Furthermore, the tighter relationship between the CV of mass-standardized DEA rates and FD than the CV of DEA rates and FD provides evidence that researchers should consider reporting mass-standardized DEA rates or some other standardized metric of DEA rates in addition to raw DEA rates.

Even though the monoculture data did not indicate that individual species stimulated DEA at different times during the season, plant FD was a strong predictor of mass-standardized DEA variability (Figure 2). These results indicate that plant communities influence microbial activity and processes and that more diverse plant communities can stabilize activity and limit variability in microbial processes. The results of this study suggest that there is a need to complement microbial community functioning data with microbial community composition data [55] to improve our understanding of how microbial diversity and ecosystem functioning are related.

## Supporting Information

Figure S1 Scatter plot of explanatory variables and DEA rates for each sample date. For every plot early May values are circles, mid-July values are triangles, and late August values are squares. A significant relationship is indicated by a * to the right of the regression line. Panels: A) Functional diversity, B) Total Inorganic N, C) MBC, D) Organic matter, and E) Soil moisture.
(0.17 MB TIF)

Figure S2 Scatter plot of explanatory variables and mass-standardized DEA rates for each sample date. For every plot early May values are circles, mid-July values are triangles, and late August values are squares. A significant relationship is indicated by a * to the right of the regression line. Panels: A) Functional diversity, B) Total Inorganic N, C) Organic matter, and D) Soil moisture.
(0.17 MB TIF)

Figure S3 The relationship between the CV of DEA rates and plant functional diversity. Scatter plot of the CV of DEA rates in early May, mid-July, and late August 2006 from each plot versus the FD value for that plot (n = 46, p = 0.28). Regression analyzed using a general linear model. Each point represents one plot.
(0.03 MB TIF)

Figure S4 DEA rates in early May, mid-July, and late August for monoculture plots. There was no significant species or date by species interaction among the monoculture plots, i.e. different species did not appear to promote different patterns in DEA over time. Monoculture plots' n for: *Carex crinita*, 2; *Carex lurida*, 1; *Scirpus cyperinus*, 2; *Juncus effusus*, 3; *Panicum virgatum*, 2; *Chasmanthium latifolium*, 1; *Eupatorium fistulosum*, 1; *Vernonia noveboracencis*, 3. Error bars indicate standard error.
(0.07 MB TIF)

## References

[1] Zedler JB (2003). Wetlands at your service: reducing impacts of agriculture at the watershed scale.. Frontiers in Ecology and the Environment.

[2] Vitousek PM, Mooney HA, Lubchenco J, Melillo JM (1997). Human domination of earth's ecosystems.. Science.

[3] Rabalais NN, Turner RE, Wiseman WJ (2002). Gulf of Mexico hypoxia, aka “The dead zone”.. Annual Review of Ecology and Systematics.

[4] Howarth RW, Sharpley A, Walker D (2002). Sources of nutrient pollution to coastal waters in the United States: Implications for achieving coastal water quality goals.. Estuaries.

[5] Groffman P, Holland E, Myrold DD, Robertson GP, Zou X, Robertson GP, Coleman DC, Bledoe CS, Sollins P (1999). Denitrification.. Standard Soil Methods for Long-Term Ecological Research.

[6] McClain ME, Boyer EW, Dent CL, Gergel SE, Grimm NB (2003). Biogeochemical hot spots and hot moments at the interface of terrestrial and aquatic ecosystems.. Ecosystems.

[7] Groffman P, Crawford MK (2003). Denitrification potential in urban riparian zones.. Journal of Environmental Quality.

[8] Groffman PM, Butterbach-Bahl K, Fulweiler RW, Gold AJ, Morse JL (2009). Challenges to incorporating spatially and temporally explicit phenomena (hotspots and hot moments) in denitrification models.. Biogeochemistry.

[9] Smith MS, Tiedje JM (1979). Phases of denitrification following oxygen depletion in soil.. Soil Biology & Biochemistry.

[10] Parsons LL, Murray RE, Smith MS (1991). Soil denitrification dynamics - Spatial and temporal variations of enzyme-activity, populations, and nitrogen gas loss.. Soil Science Society of America Journal.

[11] Pelletier F, Prevost D, Laliberte G, van Bochove E (1999). Seasonal response of denitrifiers to temperature in a Quebec cropped soil.. Canadian Journal of Soil Science.

[12] Strauss EA, Richardson WB, Cavanaugh JC, Bartsch LA, Kreiling RM (2006). Variability and regulation of denitrification in an Upper Mississippi River backwater.. Journal of the North American Benthological Society.

[13] Wallenstein MD, Peterjohn WH, Schlesinger WH (2006). Nitrogen fertilization effects on denitrifying communities and denitrification rates in an aggrading forest.. Ecological Applications.

[14] Groffman PM (1994). Denitrification in Freshwater Wetlands.. Current Topics in Wetland Biogeochemistry.

[15] Dybzinski R, Fargione JE, Zak DR, Fornara D, Tilman D (2008). Soil fertility increases with plant species diversity in a long-term biodiversity experiment.. Oecologia.

[16] Fornara DA, Tilman D (2008). Plant functional composition influences rates of soil carbon and nitrogen accumulation..

[17] Zak DR, Holmes WR, White DC, Peacock AD, Tilman D (2003). Plant diversity, soil microbial communities, and ecosystem function: Are there any links?. Ecology.

[18] Cardinale BJ, Srivastava DS, Duffy JE, Wright JP, Downing AL (2006). Effects of biodiversity on the functioning of trophic groups and ecosystems.. Nature.

[19] Yachi S, Loreau M (1999). Biodiversity and ecosystem productivity in a fluctuating environment: The insurance hypothesis.. Proceedings of the National Academy of Sciences of the United States of America.

[20] Doak DF, Bigger D, Harding EK, Marvier MA, O'Malley RE (1998). The statistical inevitability of stability-diversity relationships in community ecology.. American Naturalist.

[21] Kirby RM (1971). Soil Survey of Durham County, North Carolina: USDA Soil Conservation Service..

[22] Hall K (2003). Recommended Native Plant Species for Stream Restoration in North Carolina..

[23] Storer DA (1984). A simple high sample volume ashing procedure for determination of soil organic matter.. Communications in Soil Sciene and Plant Analysis.

[24] Maynard DG, Kalra YP, Carter MR (1993). Nitrate and Exchangeable Ammonium Nitrogen.. Soil Sampling and Methods of Analysis.

[25] Voroney RP, Winter JP, Carter MR (1993). Soil microbial biomass C and N.. Soil sampling methods of analysis.

[26] Brookes PC, Kragt JF, Powlson DS, Jenkinson DS (1985). Chloroform fumigation and the release of soil-nitrogen - The effects of fumigation time and temperature.. Soil Biology & Biochemistry.

[27] Smith MS, Tiedje JM (1979). Phases of Denitrification Following Oxygen Depletion in Soil.. Soil Biology & Biochemistry.

[28] Groffman PN, Holland EA, Myrold DD, Robertson GP, Zou X, Robertson GP, Coleman DC, Bledsoe CS, Sollins P (1999). Denitrification.. Standard Soil Methods for Long-Term Ecological Research.

[29] Moraghan JT, Buresh R (1977). Correction for Dissolved Nitrous-Oxide in Nitrogen Studies.. Soil Science Society of America Journal.

[30] Groffman PM, Tiedje JM (1989). Denitrification in north temperate forest soils - Relationships between denitrificaion and environmental factors at the landscape scale.. Soil Biology & Biochemistry.

[31] Petchey OL, Hector A, Gaston KJ (2004). How do different measures of functional diversity perform?. Ecology.

[32] Wardle DA, Bardgett RD, Klironomos JN, Setala H, van der Putten WH (2004). Ecological linkages between aboveground and belowground biota.. Science.

[33] Wardle DA (2002). Communities and Ecosystems: Linking the Aboveground and Belowground Components..

[34] Hill AR, Cardaci M (2004). Denitrification and organic carbon availability in riparian wetland soils and subsurface sediments.. Soil Science Society of America Journal.

[35] Groffman PM, Crawford MK (2003). Denitrification potential in urban riparian zones.. Journal of Environmental Quality.

[36] Hernandez ME, Mitsch WJ (2007). Denitrification potential and organic matter as affected by vegetation community, wetland age, and plant introduction in created wetlands.. Journal of Environmental Quality.

[37] Schipper LA, Harfoot CG, McFarlane PN, Cooper AB (1994). Anaerobic decomposition and denitrification during plant decomposition in an organic soil.. Journal of Environmental Quality.

[38] Kuzyakov Y (2002). Review: Factors affecting rhizosphere priming effects.. Journal of Plant Nutrition and Soil Science-Zeitschrift Fur Pflanzenernahrung Und Bodenkunde.

[39] Kuzyakov Y, Friedel JK, Stahr K (2000). Review of mechanisms and quantification of priming effects.. Soil Biology & Biochemistry.

[40] Reddy KR, Patrick WH, Lindau CW (1989). Nitrification-Denitrification at the Plant Root-Sediment Interface in Wetlands.. Limnology and Oceanography.

[41] Ettema CH, Lowrance R, Coleman DC (1999). Riparian soil response to surface nitrogen input: temporal changes in denitrification, labile and microbial C and N pools, and bacterial and fungal respiration.. Soil Biology & Biochemistry.

[42] Lowrance R, Hubbard RK (2001). Denitrification from a swine lagoon overland flow treatment system at a pasture-riparian zone interface.. Journal of Environmental Quality.

[43] Jensen CR, Luxmoore RJ, Vangundy SD, Stolzy LH (1969). Root Air Space Measurements by a Pycnometer Method.. Agronomy Journal.

[44] Petchey OL, Gaston KJ (2002). Functional diversity (FD), species richness and community composition.. Ecology Letters.

[45] Schimel J, Schulze ED, Heimann M, Harrison S, Holland E, Lloyd J (2001). Biogeochemical Models: Implicit versus Explicit Microbiology.. Global Biogeochemical Cycles in the Climate System.

[46] Tiedje JM, Simkins S, Groffman PM (1989). Perspectives on measurement of denitrification in the field including recommended protocols for acetylene based methods.. Plant and Soil.

[47] Goodroad LL, Keeney DR (1984). Nitrous-oxide emissions from soils during thawing.. Canadian Journal of Soil Science.

[48] Myrold DD (1988). Denitrification in ryegrass and winter-wheat cropping systems of western Oregon.. Soil Science Society of America Journal.

[49] Schmidt J, Seiler W, Conrad R (1988). Emission of nitrous-oxide from temperate forest soils into the atmosphere.. Journal of Atmospheric Chemistry.

[50] Liang BC, MacKenzie AF (1997). Seasonal denitrification rates under corn (Zea mays L) in two Quebec soils.. Canadian Journal of Soil Science.

[51] Weier KL, Doran JW, Power JF, Walters DT (1993). Denitrification and the dinitrogen/nitrous oxide ratio as affected by soil water, available carbon and nitrate.. Soil Science Society of America Journal.

[52] Sutton-Grier AE (2008). The role of plant functional diversity and soil amendments in regulating plant biomass and soil biogeochemistry in restored wetland ecosystems in the North Carolina Piedmont..

[53] Callaway RM, Pennings SC, Richards CL (2003). Phenotypic plasticity and interactions among plants.. Ecology.

[54] Wallenstein MD, Myrold DD, Firestone MK, Voytek MA (2006). Environmental controls on denitrifying communities and denitrification rates: insights from molecular methods.. Ecological Applications.

[55] Allison SD, Martiny JBH (2008). Resistance, resilience, and redundancy in microbial communities.. Proceedings of the National Academy of Sciences of the United States of America.

